# Monitoring training response in young Friesian dressage horses using two different standardised exercise tests (SETs)

**DOI:** 10.1186/s12917-017-0969-8

**Published:** 2017-02-14

**Authors:** Cornelis Marinus de Bruijn, Willem Houterman, Margreet Ploeg, Bart Ducro, Berit Boshuizen, Klaartje Goethals, Elisabeth-Lidwien Verdegaal, Catherine Delesalle

**Affiliations:** 1 Wolvega Equine Hospital, Stellingenweg 10, Oldeholtpade, 8474 EA The Netherlands; 2 Department of Comparative Physiology and Biometrics, Faculty of Veterinary Medicine, Ghent University, Salisburylaan 133, Merelbeke, 9820 Belgium; 3 Department of Pathobiology, Faculty of Veterinary Medicine, Utrecht University, Utrecht, The Netherlands; 4 Department of Animal Breeding and Genetics, Wageningen University, Wageningen, The Netherlands; 5 Equine Health and Performance Centre, School of Animal and Veterinary Sciences, Roseworthy Campus, University of Adelaide, Adelaide, SA 5371 Australia

**Keywords:** Friesian, Standardized exercise test, Lactic acid, Heart rate, Trot, Longitudinal

## Abstract

**Background:**

Most Friesian horses reach their anaerobic threshold during a standardized exercise test (SET) which requires lower intensity exercise than daily routine training. Aim: to study strengths and weaknesses of an alternative SET-protocol. Two different SETs (SETA and SETB) were applied during a 2 month training period of 9 young Friesian dressage horses. SETB alternated short episodes of canter with trot and walk, lacking long episodes of cantering, as applied in SETA. Following parameters were monitored: blood lactic acid (BLA) after cantering, average heart rate (HR) in trot and maximum HR in canter. HR and BLA of SETA and SETB were analyzed using a paired two-sided *T*-test and Spearman Correlation-coefficient (p* < 0.05).

**Results:**

BLA after cantering was significantly higher in SETA compared to SETB and maximum HR in canter was significantly higher in SETA compared to SETB.

The majority of horses showed a significant training response based upon longitudinal follow-up of BLA. Horses with the lowest fitness at start, displayed the largest training response. BLA was significantly lower in week 8 compared to week 0, in both SETA and SETB. A significantly decreased BLA level after cantering was noticeable in week 6 in SETA, whereas in SETB only as of week 8. In SETA a very strong correlation for BLA and average HR at trot was found throughout the entire training period, not for canter.

**Conclusions:**

Young Friesian horses do reach their anaerobic threshold during a SET which requires lower intensity than daily routine training. Therefore close monitoring throughout training is warranted. Longitudinal follow up of BLA and not of HR is suitable to assess training response. In the current study, horses that started with the lowest fitness level, showed the largest training response. During training monitoring HR in trot rather than in canter is advised. SETB is best suited as a template for daily training in the aerobic window.

**Electronic supplementary material:**

The online version of this article (doi:10.1186/s12917-017-0969-8) contains supplementary material, which is available to authorized users.

## Background

The basic principle of training is to expose a horse to a gradually increasing intensity of work. This slow and gradual build-up of workload enables the horse to adapt both physically and mentally. The latest few years, there is an increasing interest of horse riders to objectively measure fitness and training response in horses being trained. For this purpose telemetric devices are used, equipped with Global positioning system (GPS) that enable horse riders to monitor heart rate, velocity and performed distance during training [1]. When, throughout the training period these horses are subjected to a SET one can follow-up training response objectively and adjust the training schedule accordingly [2, 3]. However, little is known scientifically at this point concerning appropriate training protocols and applicable SET test protocols, for each individual sport discipline, for example dressage versus show jumping. Moreover, it can be expected that a breed specific approach is needed within each sport discipline [4–7].

Friesian horses are most commonly used for dressage and carriage driving. Especially dressage has become very popular and Friesian horses are nowadays active in high level international dressage competition such as the World Equestrian Games and the Olympics. Selective breeding on one hand and solid knowledge about proper training of these horses has become increasingly important. In a previously performed study, it was demonstrated that unlike warmblood horses, Friesian horses tend to reach their anaerobic threshold during a SET test (SETA) which requires lower intensity exercise than daily routine training. Moreover, a tendency towards familial clustering with respect to the physiological response to that SET test was reported and a genetic background for poor performance was suggested for certain Friesian breeding lines [8].

Anaerobic training on a daily basis without sufficient recovery time will not have the desired training effect. On the contrary, complications such as muscle soreness and overreaching are expected to occur in such conditions [8]. Munster et al., performed a longitudinal training follow-up of 6 weeks duration in 66 young Friesian horses selected by the Dutch Royal Friesian Studbook. These horses were subjected to a SET test (SETA) at start (week 2) and end of the training period (week 6). The first SET test encompassed solely heart rate monitoring without blood lactic acid analyses. Whereas in the SET test performed at the end of the study, both HR and blood lactic acid monitoring was performed. It was shown that many young Friesian horses reached their anaerobic lactate threshold (4 mmol/L) during SETA, that actually even represented a lower intensity exercise grade than standard daily routine training performed in the studied horses. Therefore it was suggested that Friesian horses might require a different and more careful training approach, compared to other horse breeds to prevent overreaching [8].

In the Munster study, horses were categorized as either low-, moderate-, or non-training responder based upon longitudinal follow-up. Despite the fact that all studied horses showed a positive training response, the study showed a striking heterogeneity in degree of training response between horses and the authors proposed a genetic background for their findings because of a trend of familial clustering in the type of training response seen in the study. Existence of poor athletic capability of certain Friesian breeding lines was suggested.

However, more research is needed to explore this hypothesis. The main objective of the current study was (1) to compare time profiles of HR (average and maximum) and blood lactic acid levels between the SET used in the Munster study (SET A) and an alternatively designed SET (SET B) and; (2) to evaluate training response in the studied horses and (3) to check whether HR values obtained throughout the SETs can be correlated with blood lactic acid levels. The ultimate goal of the current study was to gain more insight in order to be able to formulate advice based upon evidence based research concerning proper training of young Friesian dressage horses.

## Methods

### Study population and applied study protocol

Two different SETs (SETA and SETB) were applied throughout a 2 month training period in 9 young Friesian dressage horses (of 3 and 4 years old, and with similar training level at the start of the study). A detailed lay-out of both SETs is provided in Tables 1 and 2. SETB alternated short episodes of canter with trot and walk in both directions and thus lacked long episodes of continuous cantering as in SETA. Total cantering time was the same for both SETs.

**Table 1:** Outline of SETA

Time (min)	Exercise	Description	BLA sampling
00:00–03:00	Walk	±2 m/s	[Lactate]_start_
03:00–05:00	Left trot	left hand ±3.5 m/s	
05:00–07:00	Right trot	right hand ±3.5 m/s	
07:00–09:00	Left canter	left hand ±5 m/s	[Lactate]_canter1_
09:00–11:00	Right canter	right hand ±5 m/s	[Lactate]_canter2_
11:00–21:00	Walk	Recovery ±2 m/s	[Lactate]_end_

**Table 2:** Outline of SETB

Time (min)	Exercise	Description	BLA sampling
00:00–03:00	Walk	±2 m/s	[Lactate]_start_
03:00–05:00	Left trot	left hand ±3.5 m/s	
05:00–06:00	Left canter	left hand ±5 m/s	
06:00–07:00	Left trot	left hand ±3.5 m/s	
07:00–08:00	Left canter	left hand ±5 m/s	[Lactate]_canter1_
08:00–09:00	Walk	left hand ± 2 m/s	
09:00–11:00	Right trot	right hand ±3.5 m/s	
11:00–12:00	Right canter	right hand ±5 m/s	
12:00–13:00	Right trot	right hand ±3.5 m/s	
13:00–14:00	Right canter	right hand ±5 m/s	[Lactate]_canter2_
14:00–24:00	Walk	Recovery ±2 m/s	[Lactate]_end_

All participating horses were clinically examined and checked for absence of lameness before the start of the study. Throughout the training period, the riders were asked to daily record appetite, and possible presence of adverse signs such as lameness, disease or reluctance to work in a logbook.

All horses were subjected to the same training protocol, comprising of 30–45 min of basic dressage training comparable to the workload in SETB, three times per week. All horses were subjected to SETA (at day 1 of week 0, 2, 4, 6, and 8) (See Table 1 for SET layout) and SETB (at day 5 of week 0, 2, 4, 6 and 8) (See Table 2 for SET layout), during which HR (GPS equipped Polar RC 3) and BLA (Lactate Pro hand held analyzer) were monitored throughout the SETs. For each SET these parameters were monitored at following time points: BLA after cantering, average HR in trot and maximum HR in canter. Blood was collected by briefly stopping the horses during the SET and collecting a blood sample from the jugular vein in a plain syringe. Blood was collected at the start of training [Lactate]_start_, directly after the left canter [Lactate]_canter1_ and right canter [Lactate]_canter2_ and at the end [Lactate]_end_ during SET A and B. Time points of blood collection are marked [Lactate] in Tables 1 and 2. Plasma lactate concentrations were measured with a hand held analyzer: Akray Lactate pro. Lactate concentrations <0.8 mmol/L were below the detection limit and were set at 0.8 mmol/L. Ambient temperature and humidity were recorded during the measurements [9, 10].

The horse rider was equipped with a polar watch to enable him/her to control speed during SET tests.

### Data processing and statistical analysis

The following heart rate parameters were calculated from the Polar heart rate curves for both SETs (SETA and SETB): average heart rate during trot (SETA: HRav_trot_, SETB: HRav_trot1,_ HRav_trot2,_ HRav_trot3_ and HRav_trot4_), maximum heart rate during left canter (HR_maxLC_) and maximum heart rate during right canter (HR_maxRC_). For SETA, also average heart rate during left canter (HR_LC_) and right canter (HR_RC_) were calculated. BLA at the beginning of the set was recorded as [Lactate]_start_, after left canter as [Lactate]_canter1_, after right canter as [Lacate]_canter2_ and at the end of the SET as [Lactate]_end_.

Comparisons between HR and BLA of SETA and SETB were analyzed using a paired two-sided *T*-test adjusted with a Tukey-Kramer correction. Correlations between HR and BLA were analyzed using a Spearman Correlation Coefficient. All analyses were performed in SAS version 9.4 (SAS Institute., USA). *P* values less than 0,05 were considered significant. A Spearman Correlation Coefficient higher than 0.6 was considered a strong correlation in this study.

All nine horses were categorized as clinically healthy, without any sign of lameness or illness and remained that way throughout the study. Two horses were lost to follow up in the last training week due to circumstances. Ambient temperature during the study ranged between 10 and 18 °C. Relative humidity ranged between 75 and 87%.

## Results

### Comparison BLA and HR in SETA versus SETB

**Fig. 1:**
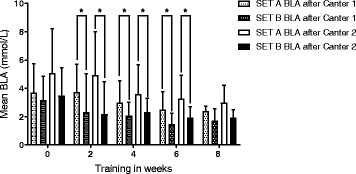
BLA concentration difference between SETA and SETB. Mean BLA and SD are depicted and significant differences (*P* < 0.05) are marked by an *asterix*. Analysis of the BLA in SETA and SETB revealed that the blood lactic acid time profile significantly differed between the two different SETs. Mean BLA levels after canter1 and after canter 2 were significantly higher in week 2, week 4 and week 6 in SETA, in which the horses canter nonstop during 4 min, when compared to SETB, in which the workload was similar but more gait variation was applied

BLA levels at start of the SETs were less than 0.8 mmol/L for every horse in every week during the study. Analysis of the BLA in SETA and SETB revealed that the blood lactic acid time profile significantly differed between the two different SETs (see Fig. 1). Mean BLA levels [Lactate]_canter1_ and [Lactate]_canter2_ were significantly higher in SETA, in which the horses canter nonstop during 4 min (see Table 1), when compared to SETB, in which the workload was similar but more gait variation was applied in week 2, week 4 and week 6 (see Fig. 1). Blood [Lactate]_end_, after 10 min of walking at the end of each SET didn’t differ significantly between SETs at any week.

**Fig. 2:**
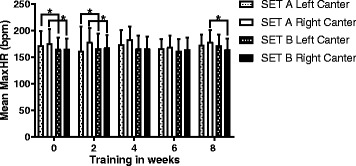
Maximum Heartrate difference between SETA and SETB. Mean of the maximum HR and SD are depicted and significant differences (*P* < 0.05) are marked by an *asterix*. Maximum HR during left canter and during right canter was significantly higher in SETA in week 0 and week 2 and for right canter in week 8. In week 4 and 6 no significant difference in maximum HR during canter was observed

The mean HR_maxLC_ in SET A was 172 ± 23 beats/min and in SETB was 165 ± 23 beats/min. Mean HR_maxRC_ in SETA was 177 ± 24beats/min and 165 ± 20 beats/min in SETB. Maximum HR during left canter and during right canter was significantly higher in SETA in week 0 and week 2 and for right canter in week 8 (see Fig. 2). In week 4 and 6 no significant difference in maximum HR during canter was observed.

The smaller intervals of cantering performed in SETB lead to lower heart rate peaks. Mean heart rate in trot was not significantly different between SETA and SETB (130 ± 14 beats/min).

### Training response–blood lactic acid time profile

**Fig. 3:**
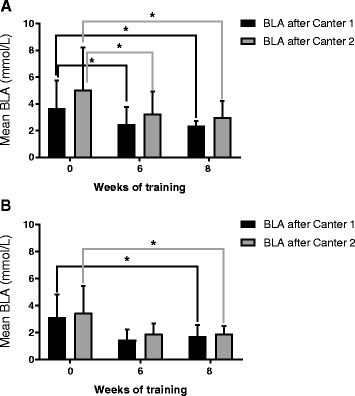
Training response assessed by SETA and SETB. Training response was shown by comparing SET BLA results of week 8 with BLA in week 0. SET BLA levels after both canter 1 and after canter 2 are shown. Mean BLA and SD are depicted and significant differences (*P* < 0.05) are marked by an *asterix*. Both SETs were compared and it was shown that SETA could distinguish a positive training response sooner, already in week 6, than SETB, which could only show a positive training response in week 8

The training response is reflected by a decrease in BLA concentration during SETS over the 8 week training period. In week 0 mean BLA [Lactate]_canter1_ was 3.9 ± 2.1 mmol/L and 3.5 ± 1.5 mmol/L in respectively SETA and SETB. In week 8 mean BLA after canter1 was significantly lower 2.4 ± 0.5 mmol/L in SET A and 1.7 ± 0.8 mmol/L in SET B (borderline significant). A similar response was expressed in BLA [Lactate]_canter2_ with mean lactate concentrations of 5.0 ± 3.2 mmol/L and 3.8 ± 1.9 mmol/L in week 0 compared to the significantly lower 3.0 ± 1.2 mmol/L and 1.9 ± 0.6 mmol/L in week 8 (see Fig. 3). Mean BLA [Lactate]_end_ also decreased in both SETs: 2.7 ± 2.0 mmol/L and 1.3 ± 0.7 mmol/L in week 0 compared to 0.9 ± 0.2 mmol/L and 0.9 ± 0.2 mmol/L in week 8.

Seven out of 9 horses showed a significant positive training response and this response could only be assessed by means of longitudinal follow-up of [Lactate]_canter1_ and [Lactate]_canter2_ (See Table 3).

**Table 3:** Overview of percentage of horses showing a positive training response. The training response is defined by a significant decrease in BLA during SETs after 8 weeks of training

SET	Blood sampling moment	% of horses showing positive training response	% of horses showing no positive training response
SETA	BLA canter 1	67	33
	BLA canter 2	78	22
SETB	BLA canter 1	89	11
	BLA canter 2	89	11

Interestingly, in the current study horses with the highest blood lactic acid concentration during SETs at the start of the study displayed the largest training response.

SETA was more suitable to early assess training response. A significantly decreased BLA level after cantering was already noticeable in week 6 in SETA, whereas in SETB only as of week 8.

### Training response–heart rate time profile

Throughout the trial in none of the horses a significant decrease in average or maximum HR was found, not for SET A, nor for SET B. Only trends were seen. The heart rate time profile did show a moderate decrease, however not as consistent as the lactate parameters. In **SETA** results for HR_maxLC_ and HR_maxRC_ showed a slightly lower mean in week 8 compared to week 0. HR_maxLC_ was 177 ± 30 beats/min in week 0 and 174 ± 19 beats/min in week 8. HR_maxRC_ was 182 ± 28 beats/min in week 0 and 179 ± 22 beats/min in week 8.

Results were similar for HR_LC_ (169 ± 30 beats/min in week 0 and 165 ± 19 beats/min in week 8) and HR_RC_ (173 ± 29 beats/min in week 0 and 171 ± 21 beats/min in week 8), also not significant. Mean HR_trot_ was lower in week 8 than in week 0 (132 ± 14 beats/min and 136 ± 22 beats/min) however the difference was not significant.

In **SETB** HR_maxLC_ was slightly higher in week 8 (165 ± 24 beats/min) compared to week 0 (167 ± 19 beats/min). HR_maxRC_ did show a mild decrease from 166 ± 21 beats/min in week 0, to 164 ± 20 beats/min in week 8. Mean HR_trot_ decreased from 131 ± 14 beats/min to 130 ± 12 beats/min. In SETB none of the heart rate parameters showed a significant training response which was comparable the HR results presented for SETA.

### Correlations between heart rate and blood lactic acid for different SETs and gaits

Correlations between HR parameters and BLA [Lactate]_canter1_, [Lactate]_canter2_ at start and at the end of both SETA and SETB were studied in week 0, 2, 4, 6 and 8.

**Fig. 4:**
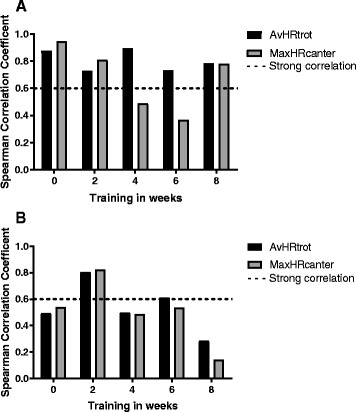
Correlation between BLA and HR for SETA and SETB. Correlations between average HR during trotting or maximum HR while cantering and BLA after cantering are shown for weeks 0, 2, 4, 6 and 8. The correlation between BL and HR is shown for both SETA in panel (**a**) and SETB in panel (**b**). A Spearman Correlation Coefficient higher than 0.6 is considered a strong correlation in this study. It was shown that throughout the training period the HR in trot was strongly correlated to the BLA values after cantering. For SETA this effect was more pronounced than in SETB

For SETA the correlations between HR in every gait and BLA[Lactate]_canter2_ are very strong in week 0 (Fig. 4 panel A). Results of the analysis of BLA after [Lactate]_canter2_ and the maximum and average HRs throughout SETA are shown in Fig. 4 panel a and in Additional file 1: Table S1. For SETA, only for trot significant correlations were found throughout the complete study. As depicted in Fig. 4 Panel a there was a strong correlation between HR in trot (HR_trot_) with 1) BLA [Lactate]_canter2_, with 2) BLA [Lactate]_canter1_ (data not shown) and with 3) BLA [Lactate]_end_ (data not shown).

Correlations were analyzed between HR parameters and 1) BLA [Lactate]_canter1_ and 2) BLA [Lactate]_end_ for SETA and SETB, and comparable results were found.

The correlation of BLA [Lactate]_canter2_ with HR parameters becomes less strong in week 2 and even disappears in week 4 and 6. However, in week 8 this correlation is strong again.

In SETB there were only a few strong correlations found between BLA and HR parameters and existing correlations were all weaker than those found in SETA, as is shown in Fig. 4, panel b and Additional file 1: Table S2. For SETB only in week 2 a consistent strong correlation was found between all HR parameters and BLA [Lactate]_canter2_. In the other training weeks there was no strong correlation present, except for week 6 (HRav_trot3_ and BLA [Lactate]_canter2_).

Importantly, BLA and average HR at trot have a very strong correlation throughout the entire training period (see Fig. 4, panel a), which was not the case for canter. This correlation was more pronounced and present throughout a longer period in SETA when compared to SETB (see Fig. 4, panels a and b).

## Discussion

In the current study, again, most Friesian horses crossed their anaerobic threshold when performing SETA. And so, this finding is in accordance with previous reports, suggesting that Friesian horses reach their anaerobic threshold quicker than other horse breeds [8]. Other research encompassing anaerobic lactate threshold showed that most horses do not reach their anaerobic threshold at velocities reached in a standard canter like the one used in SETA [11–13]. Friesian horses are renowned for their impressive trot with high knee action, however, cantering is a much more difficult gait for them. In that respect, the relatively long four minutes of continuous canter applied in SETA might cause the young horses to reach their anaerobic threshold of 4 mmol/L much earlier. Another possible explanation as to why Friesian horses tend to cross their anaerobic threshold already at low intensity exercise, is their muscle fibre composition. It is possible that Friesian horses, just like Quarter horses predominantly possess fast twitch fibers in most important locomotor muscles [7]. Up until now, no study has been performed on muscle fibre typing in Friesian horses.

The horses in this study had higher blood lactate concentrations and higher peak heart rates in SETA when compared to SETB. As mentioned previously, SETB alternated short episodes of canter with trot and walk in both directions and thus lacked long episodes of continuous cantering as in SETA. Total cantering time was the same for both SETs. It is to be expected that shorter cantering intervals limit lactate accumulation and heart rate peaks. Indeed, the frequent changes in gait and directions incorporated into SETB, allow for intermittent recuperation. Our findings show that SETB provides a good template for training young Friesian dressage horses in the aerobic window. It resembles the approach of interval training, which essentially entails conditioning by applying frequent alternates between varying degrees of effort and incorporating periods of recovery in between efforts. This approach trains the equine body to efficiently switch between aerobic and anaerobic pathways [14, 15]. SETA and SETB were always performed with a 2 days interval, but always in the same order: SET B was always preceded by SETA, unfortunately no block design was applied.

Both SETA and SETB were suitable to assess training response in the studied horses, however SETA had a greater discriminative value than SETB with that respect, since a distinct training response could already be identified as of week 6 in SETA, whereas in SETB only as of week 8. Both BLA levels and HRs decreased progressively during both SETs over the course of the 8 weeks of training. However, this decrease was only significant for BLA. These results show that training response can only be properly assessed by longitudinal follow-up of BLA values. This is in accordance with previous research. [12 Several studies have shown that aerobic capacity in horses can improve without significant heart rate decrease as an effect of training [11, 16–19]. Moreover, HR is not only influenced by effort, but even so by emotional excitability, reason for which many studies on equine welfare have included the follow up of heart rate variability in their study design [20, 21]. In the study performed by Munsters et al., no BLA levels were determined at start of the training study, only a longitudinal follow up of HRs was performed.

However, BLA level determination requires blood collection, and it is not feasible to advocate this during each training day. Heart rate monitoring is a non-invasive and elegant technique and the results of our study show a high and consistent correlation between BLA levels after cantering and average HR at trot, which was not the case for average HR at canter. This finding could support the hypothesis that canter is quite a difficult and energy demanding gait for Friesian horses. The fact that canter is an exciting event for horses and not only an effort probably partly explains why correlations between BLA and HR at canter are less consistent. And the current study only encompasses 9 horses. However, the strong correlation between average HR at trot and BLA after canter in all 9 Friesian horses suggests that longitudinal follow-up of HR during trot is an interesting approach to monitor training response of young Friesian dressage horses. Munsters et al. found a significant correlation between mean HR in canter and BLA after canter [8]. Munoz et al. found significant correlations between HR, BLA and stride length in canter in Andalusian horses but not in Anglo-Arabian and Arabian horses [16]. The results of our study suggest that HR in trot is a useful parameter to monitor while training Friesian horses. However, more research is needed to further explore this approach.

Striking in our study is the fact that horses with the worst training parameters at the beginning of the training, also showed the largest training response. Horses that had the highest heart rates and blood lactic acid concentrations in week 0, also showed the largest response to training. The results of our study thus show that caution is warranted when categorizing a young Friesian horse as predisposed to poor performance and thus unfit for a professional sports career, based upon poor parameter results obtained during a SET performed at start of the training. Our study also shows that the duration of follow-up is important and that depending on the used SET, the necessary duration of follow-up differs. In our study this was 6 weeks when using SETA and 8 weeks when using SETB.

Our study population was too small to assess possible influence of pedigree on training response. And keeping in mind the very narrow genetic basis of the Friesian breed, such studies would entail the need for a very large study population of several of hundreds of horses.

## Conclusion

Young Friesian horses do reach their anaerobic threshold during a SET test which requires lower intensity than daily routine training. Therefore close monitoring throughout training is warranted. Longitudinal follow up of BL and not of HR is suitable to assess training response and necessary duration of follow up depends on the applied SET test. SETA has the highest value to follow-up training response, whereas SETB provides a solid template to train Friesian horses in the aerobic window. Monitoring HR in trot and incorporating longer periods of trot during training of young Friesian horses is advised. Finally, caution is warranted to categorize a young Friesian dressage horse as unfit for a sportive career based upon poor SET parameter results at start of the training.

## Additional file

Additional file 1: Table S1. Correlation between heart rate and [Lactate]_canter2_ for SETA. **Table S2.** Correlation between HR and [Lactate]_canter2_ for SETB_._ *Correlations heart rate and blood lactic acid*. Correlations between HR parameters and BLA [Lactate]_canter1_, [Lactate]_canter2_ at start and at the end of both SETA and SETB were studied in week 0, 2, 4, 6 and 8. Results of the analysis of BLA after [Lactate]_canter2_ and the maximum and average HRs throughout SETA are shown in supplementary Table 1. Results of the analysis of BLA after [Lactate]_canter2_ and the maximum and average HRs throughout SETB are shown in supplementary Table 2. Significant correlation coefficients are marked by an asterix (*). (PDF 22 kb)

## Abbreviations

BLA: Blood lactic acid
GPS: Global positioning system
HR: Heart rate
HR_avTrot_: Average heart rate during trot
HR_LC_: Average heart rate during left canter
HR_maxLC_: Maximum heart rate during left canter
HR_maxRC_: Maximum heart rate during right canter
HR_RC_: Average heart rate during right canter
SET: Standardized exercise test
SETA: Standardized exercise test A
SETB: Standardized exercise test B
