# Supplementary material for: Monitoring training response in young Friesian dressage horses using two different standardised exercise tests (SETs)
Source: BMC Vet Res. 2017 Feb 14;13:49. doi: 10.1186/s12917-017-0969-8 (PMC5309987; doi:10.1186/s12917-017-0969-8)
Supplement: Additional file 1: Table S1. — Correlation between heart rate and [Lactate]canter2 for SETA. Table S2. Correlation between HR and [Lactate]canter2 for SETB. Correlations heart rate and blood lactic acid. Correlations between HR parameters and BLA [Lactate]canter1, [Lactate]canter2 at start and at the end of both SETA and SETB were studied in week 0, 2, 4, 6 and 8. Results of the analysis of BLA after [Lactate]canter2 and the maximum and average HRs throughout SETA are shown in supplementary Table 1. Results of the analysis of BLA after [Lactate]canter2 and the maximum and average HRs throughout SETB are shown in supplementary Table 2. Significant correlation coefficients are marked by an asterix (*). (PDF 22 kb) [file 12917_2017_969_MOESM1_ESM.pdf]

Supplementary table 1: Correlation between heart rate and BLA [Lactate]$_{canter2}$ for SETA

An asterix indicates a significant correlation (P<0.05).

Weeks	**HR parameter**	HR_{maxLC}	HR_{maxRC}	HR_{avLC}	HR_{avRC}	$HRav_{trot}$
0	**Correlation to [Lactate]$_{canter2}$**	0.96235 P <0.0001*	0.92888 P=0.0003*	0.96235 P<0.0001*	0.96235 P=0.0003*	0.87867 P=0.0018*
2	**Correlation to [Lactate]$_{canter2}$**	0.778571 P= 0.0229*	0.83834 P=0.0093*	0.74096 P=0.0354*	0.77846 P=0.0229*	0.72996 P=0.0256*
4	**Correlation to [Lactate]$_{canter2}$**	0.53333 P=0.1392	0.44352 P=0.2318	0.75314 P=0.0191*	0.51667 P=0.1544	0.89541 P=0.0011*
6	**Correlation to [Lactate]$_{canter2}$**	0.17573 P=0.6511	0.45000 P=0.2242	0.31799 P=0.4043	0.42018 P=0.2602	0.73333 P=0.0246*
8	**Correlation to [Lactate]$_{canter2}$**	0.77481 P=0.0408*	0.78571 P=0.0362*	0.78571 P=0.0362*	0.85714 0.0137*	0.78571 P=0.0362*

Supplementary table 2: Correlation between HR and BLA [Lactate]$_{canter2}$ for SETB

An asterix indicates a significant correlation (P<0.05).

Weeks	**HR parameter**	HR_{maxLC1}	HR_{maxLC2}	HR_{maxRC1}	HR_{maxRC2}	$HRav_{trot1}$	$HRav_{trot2}$	$HRav_{trot3}$	$HRav_{trot4}$
0	**Correlation to [Lactate]$_{canter2}$**	0.51498<br>P =0.1915	0.48536<br>P=0.1854	0.58333<br>P=0.0992	0.57985<br>P=0.1017	0.50210<br>P=0.1684	0.47619<br>P=0.2329	0.50000<br>P=0.1705	0.40168<br>P=0.2839
2	**Correlation to [Lactate]$_{canter2}$**	0.77846<br>P= 0.0229*	0.80241<br>P=0.0165*	0.82636<br>P=0.0114*	0.88624<br>P=0.0034*	0.78916<br>P=0.0199*	0.80241<br>P=0.0165*	0.82636<br>P-0.0114*	0.86229<br>P=0.0059*
4	**Correlation to [Lactate]$_{canter2}$**	0.47060<br>P=0.2011	0.50422<br>P=0.1663	0.54624<br>P=0.1281	0.42018<br>P=0.2602	0.50422<br>P=0.1663	0.50422<br>P=0.1663	0.48741<br>P=0.1832	0.56963<br>P=0.1094
6	**Correlation to [Lactate]$_{canter2}$**	0.45000<br>P=0.2242	0.45000<br>P=0.2242	0.65000<br>P=0.0581	0.60000<br>P=0.0876	0.50000<br>P=0.1705	0.60000<br>P=0.0876	0.73641<br>P=0.0237*	0.56067<br>P=0.1163
8	**Correlation to [Lactate]$_{canter2}$**	-0.07229<br>P=0.8649	0.24098<br>P=0.5653	0.20000<br>P=0.6349	0.19279<br>0.6474	0.38557<br>P=0.3455	0.21688<br>P=0.6059	0.25303<br>P=0.5454	0.27879<br>P=0.5037
